# A 20-year study of autoimmune polyendocrine syndrome type II and III in Taiwan

**DOI:** 10.1530/ETJ-23-0162

**Published:** 2023-11-23

**Authors:** Hsu-Hua Tseng, Yen-Bo Lin, Kuan-Yu Lin, Chia-Hung Lin, Hung-Yuan Li, Chia-Hsuin Chang, Yi-Ching Tung, Pei-Lung Chen, Chih-Yuan Wang, Wei-Shiung Yang, Shyang-Rong Shih

**Affiliations:** 1 Department of Internal Medicine, National Taiwan University Hospital, Taipei, Taiwan; 2 Division of Endocrinology and Metabolism, Department of Internal Medicine, National Taiwan University Hospital, Bei-Hu Branch, Taipei, Taiwan; 3 Division of Endocrinology and Metabolism, Department of Internal Medicine, National Taiwan University Hospital, Yun-Lin Branch, Douliu City, Taiwan; 4 Division of Endocrinology and Metabolism, Department of Internal Medicine, National Taiwan University Hospital, Hsin-Chu Branch, Hsin-Chu, Taiwan; 5 Division of Endocrinology and Metabolism, Department of Internal Medicine, National Taiwan University Hospital, Taipei, Taiwan; 6 Department of Pediatrics, National Taiwan University Hospital, Taipei, Taiwan; 7 Graduate Institute of Medical Genomics and Proteomics, National Taiwan University College of Medicine, Taipei, Taiwan; 8 Department of Medical Genetics, National Taiwan University Hospital, Taipei, Taiwan; 9 Department of Internal Medicine, National Taiwan University College of Medicine, Taipei, Taiwan; 10 Graduate Institute of Clinical Medicine, National Taiwan University College of Medicine, Taipei, Taiwan; 11 Center of Anti-Aging and Health Consultation, National Taiwan University Hospital, Taipei, Taiwan

**Keywords:** autoimmune polyendocrine syndrome, primary adrenal insufficiency, type 1 diabetes mellitus, autoimmune thyroid disease, anti-parietal cell antibody

## Abstract

**Purpose:**

Autoimmune polyendocrine syndrome (APS) is a rare immune-endocrinopathy characterized by the failure of at least two endocrine organs. Clinical characteristics have mainly been described in the Western population. This study comprehensively analyzed the demographic and clinical manifestations of APS II and APS III in Taiwan.

**Methods:**

Patients aged ≥20 years with a diagnosis of APS II or APS III in ten hospitals between 2001 and 2021 were enrolled. The clinical and serological characteristics of the patients were retrospectively reviewed.

**Results:**

Among the 187 enrolled patients (45 men and 142 women); only seven (3.7%) had APS II, while the others had APS III. Fifty-five patients developed hyperthyroidism and 44 patients developed hypothyroidism. Men were diagnosed with APS at a younger age than women (16.8 vs 27.8 years old, *P* = 0.007). Most patients were initially diagnosed with type 1 diabetes mellitus. There was a positive correlation between age at diagnosis and the likelihood of developing thyroid dysfunction. For every year older patients were diagnosed with APS III, the risk of developing hyperthyroidism increased by 3.6% (*P* = 0.002), and the risk of developing hypothyroidism increased by 3.7% (*P* = 0.035). Positive anti-parietal cell antibodies (APCA) were associated with a higher risk of anemia in patients with APS III (*P* < 0.001).

**Conclusion:**

This study provides the most comprehensive analysis of APS II and APS III in Asia. The percentage of patients with APS II was significantly lower than in the Western population. A second autoimmune endocrinopathy may develop several years after the first one. APCA examination is valuable when evaluating anemia in patients with APS.

## Introduction

Autoimmune polyendocrine syndrome (APS) was initially described in France by Claude and Gourgerot in 1908 (1). It is characterized by immunopathological involvement of at least two endocrine organs, such as the adrenal gland, thyroid, and pancreas (2). APS frequently coexists with some nonendocrine diseases, including vitiligo, alopecia, celiac disease, and autoimmune gastritis (AIG) (3). The prevalence of APS ranges widely from 1:1000 to 1:20,000 in the general population (4, 5). In 1980, Neufeld *et al.* proposed the initial clinical classification of APS (3), categorizing it into four types: a less common juvenile type I and the more prevalent adult types II to IV. APS type I (APS I) is a monogenetic autosomal recessive inherited syndrome with mutations in the autoimmune regulator gene (*AIRE*), characterized by chronic mucocutaneous candidiasis, primary adrenal insufficiency (PAI), and autoimmune hypoparathyroidism (6, 7, 8, 9). In contrast, the inheritance pattern of adult APS types is polygenic, involving several specific genes associated with human leukocyte antigen (HLA), cytotoxic T-lymphocyte-associated protein 4, and protein tyrosine phosphatase nonreceptor type 22, which have been linked to APS (10, 11, 12). APS type II (APS II) is defined by the presence of PAI and at least one of the following endocrinopathies: type 1 diabetes mellitus (DM) (T1DM) or autoimmune thyroid disease (AIT). APS type III (APS III) is defined by the coexistence of AIT with another autoimmune endocrine disorder, such as T1DM, while the adrenal gland remains uninvolved. APS type IV (APS IV) is identified by the presence of two or more organ-specific autoimmune endocrinopathies that do not fall into the definitions of APS type I to III (3, 13). In 2019, Frommer *et al.* adjusted the definition of APS III, excluding PAI and published it (2). According to their revised classification, APS III is defined as the presence of T1DM and AIT without affecting the adrenal gland; therefore, PAI was excluded. Conversely, Eisenbarth *et al.* proposed an alternative APS classification in 2004, combining APS II and APS III into a single category, APS II (14). Some researchers supported this simple classification, arguing that there was insufficient evidence to support distinct causes of the two subtypes; therefore, splitting this syndrome into further subtypes was unnecessary (4).

In the present study, we employ the four-type classification of APS adjusted by Frommer *et al.* to provide a comprehensive description and analysis. Based on this classification, APS II is defined as the coexistence of PAI along with either AIT or T1DM, while APS III is characterized by the presence of AIT and T1DM without PAI (2). We hypothesize that there may exist variations in both the clinical presentation and pathogenesis among distinct adult subtypes of APS (15). It is worth noting that the majority of published studies have predominantly focused on Western countries, with limited research dedicated to investigating APS within the Asian population (16, 17, 18, 19). Therefore, in our study, we specifically aim to examine the clinical and serological characteristics of APS II and APS III within Taiwan.

## Materials and methods

### Study population

We conducted a retrospective analysis of patients aged 20 years or older who were diagnosed with APS II or APS III in ten hospitals in Taiwan, which include the National Taiwan University Hospital (NTUH), National Taiwan University (NTU) Cancer Center, NTU Children’s Hospital, NTUH Bei-Hu branch, NTUH Jin-Shan Branch, three afﬁliated hospitals in the NTUH Hsin-Chu branch, and two afﬁliated hospitals in the NTUH Yun-Lin branch. The potential study participants were screened and obtained through the National Taiwan University Hospital-Integrated Medical Database (NTUH-iMD) for the period spanning from 2001 to 2021 (20).

## Methods

The diagnosis of APS II was defined as the presence of PAI along with either T1DM or AIT, or both, whereas the diagnosis of APS III was defined as the presence of AIT and T1DM without PAI (2, 21). Patients with APS I or APS IV were excluded from the study.

The diagnosis of PAI was made by the associated symptoms and signs, low serum cortisol levels, and elevated plasma adrenocorticotropic hormone (ACTH) levels. ACTH stimulation test was performed to confirm the diagnosis when necessary (22, 23). Patients with PAI caused by nonautoimmune etiologies such as adrenalectomy, infection, and malignancies were excluded. Rare genetic disorders causing childhood PAI were also excluded from the medical history. Adrenal antibodies were not checked in the present study because it was not available in our hospital system.

T1DM was diagnosed in patients with hyperglycemia meeting the diagnostic criteria of DM, confirmed poor islet cell reserve by the glucagon test, and the presence of autoantibodies against glutamic acid decarboxylase or tyrosine phosphatase if available (22). Those with type 2 DM, gestational DM, or other types of DM were excluded.

Overt hyperthyroidism is defined as elevated serum concentrations of thyroid hormones with suppressed serum thyroid-stimulating hormone (TSH) levels. Subclinical hyperthyroidism is defined as a low serum TSH concentration and normal thyroid hormone levels. In contrast, overt hypothyroidism was defined as decreased thyroid hormone and increased TSH levels. Subclinical hypothyroidism is defined as elevated serum TSH and normal thyroid hormone levels (16). Patients with transient hyperthyroidism followed by hypothyroidism would be classified into the hypothyroidism group. Patients with Graves’ hyperthyroidism followed by hypothyroidism would be classified into the hyperthyroidism group.

The diagnosis of AIT was made in patients with at least one positive thyroid autoantibody, including anti-thyroid peroxidase antibody (anti-TPO Ab, reference range: <5.61 IU/mL, Abbott Architect i2000SR using reagents provided by Abbott Diagnostics), anti-thyroglobulin antibody (anti-Tg Ab, reference range: <14.3 IU/mL, Abbott Architect i2000SR using reagents provided by Abbott Diagnostics), and thyrotropin-binding inhibitory immunoglobulin (TBII, reference range: < 10%, R.S.R. Ltd. Cardiff, UK). TBII indicates the amount of TSH receptor antibodies (TSHRAb) and was measured using radioimmunoassay (24). According to the manufacturer’s protocol, the patient’s serum was added to a tube coated with the TSH receptor. TSHRAb in serum inhibits the binding of radiolabeled porcine TSH to the coated tube. After washing out the fluid, the radioactivity of the tube was measured. Lower radioactivity indicated higher serum TSHRAb levels. The value was calculated using the following formula: TBII = 1 − (*B*/*B*_0_), where *B* represents the gamma radiation count in the patient’s serum, and *B*_0_ is the gamma radiation count of the negative control (24). Anti-parietal cell antibodies (APCA) are associated with AIG, which is a comorbidity of APS (25). Therefore, we also recorded the serum APCA levels (reference range: < 1:20, Euroimmun IIFT: Stomach (Monkey), Germany).

We reviewed the medical records and documented the time sequence of diagnosis based on the APS criteria of each patient. The first disease diagnosed was defined as the first diagnosis documented in the medical record. If two diseases were diagnosed within 1 month, they were labeled as ‘diseases diagnosed at almost the same time’. We also recorded the patients’ laboratory data.

### Statistical analysis

Continuous variables with normal distribution were presented as mean ± standard deviation (SD), while non-normally distributed variables were presented as medians (interquartile ranges (IQRs)). The Student’s *t*-test was used to compare means between groups with normally distributed datasets, and the Wilcoxon–Mann–Whitney test was used if the dependent variables were non-normally distributed. Categorical variables were compared using the chi-squared test or Fisher’s exact test. The Kaplan–Meier method was used to estimate the cumulative rate of patients developing overt hyperthyroidism, subclinical hyperthyroidism, overt hypothyroidism, and subclinical hypothyroidism, since positive thyroid autoantibodies were detected.

A logistic regression analysis was performed to analyze the relationship between the age at APS diagnosis and the likelihood of developing hyperthyroidism or hypothyroidism in patients with APS III. The linearity of continuous variables concerning the dependent variable was checked. Odds ratios (OR) and the associated 95% confidence intervals (CIs) were also calculated. Statistical significance was set at a two-tailed *P* < 0.05. Stata/SE 14.0 for Windows (StataCorp. LP, College Station, TX, USA) was used for statistical analyses.

## Results

This study comprised 187 patients, with 45 (24.1%) men and 142 (75.9%) women. The summarized data on the first relevant disease, thyroid autoantibody concentrations, and thyroid function status can be found in Table 1. Notably, male patients were diagnosed with APS at a younger age compared to female patients (*P* = 0.007). T1DM was the most frequent first diagnosis, while AIT was hardly the first diagnosed disease in men (*n* = 0) compared with women (*n* = 25). Among the participants, 55 patients developed hyperthyroidism, with 51 experiencing overt hyperthyroidism and 4 having subclinical hyperthyroidism. On the other hand, 44 patients developed hypothyroidism, including 17 overt hypothyroidism and 27 subclinical hypothyroidism cases. Five patients with subclinical hypothyroidism experienced short-term hyperthyroidism precedingly. Remarkably, none of the patients with hyperthyroidism experienced non-iatrogenic hypothyroidism during the follow-up periods. Furthermore, gender differences were observed in the thyroid function status (*P* = 0.046).

**Table 1:** Basic characteristics of the patients (*n* = 187). Data are presented as *n* (%) or as median (IQR).

	Male	Female	*P*^a^
*n* (%)	45 (24.1)	142 (75.9)	
Age of APS diagnosed (years)	16.8 (9.6–29.9)	27.8 (15.6–39.5)	**0.007**
Types of APS			0.675
Type II	2 (4.4)	5 (3.5)	
Type III	43 (95.6)	137 (96.5)	
Disease first diagnosed among T1DM, AIT, and PAI^b^			**0.002**
T1DM	27 (61.4)	82 (59.4)	
AIT	0	25 (18.1)	
PAI	1 (2.3)	2 (1.4)	
T1DM and AIT at almost the same time	16 (35.6)	31 (21.8)	
PAI and AIT at almost the same time	1 (2.3)	1 (0.7)	
Thyroid autoantibody concentrations^c^			
TBII (%)	34.4 (9.1–62.6)	24.7 (7.1–59.5)	0.518
Anti-TPO Ab (IU/mL)	266.5 (85.5–995)	384.9 (61.1–1465.8)	0.450
Anti-Tg Ab (IU/mL)	65.7 (8.7–297.4)	59.3 (8.2–376.6)	0.966
Thyroid function^d^			**0.046**
Overt hyperthyroidism	7 (15.6)	44 (31.0)	
Subclinical hyperthyroidism	0	4 (2.8)	
Overt hypothyroidism	5 (11.1)	12 (8.5)	
Subclinical hypothyroidism	8 (17.8)	19 (13.4)	
Euthyroid	25 (55.6)	63 (44.4)	

^a^Comparison between female and male; bold values denote statistical significance at the *P* < 0.05 level;^b^Cannot be identified from the chart review of one patient; ^c^TBII was available in 74 patients, anti-TPO Ab was available in 186 patients, and anti-Tg Ab was available in 172 patients; ^d^Refers to thyroid function before data cutoff date, excluding iatrogenic causes such as operation, radioiodine, and antithyroid medication or levothyroxine overused.

AIT, autoimmune thyroid disease; anti-TPO Ab, anti-thyroid peroxidase antibody; APS, autoimmune polyendocrine syndrome; Anti-Tg Ab, anti-thyroglobulin antibody; PAI, primary adrenal insufficiency; T1DM, type 1 diabetes mellitus; TBII, thyrotropin-binding inhibitory immunoglobulin.

The majority of patients (96.3%) were diagnosed with APS III, whereas seven patients were classified as having APS II as shown in Table 2. Patients with APS III were significantly younger at diagnosis compared to those with APS II (*P* = 0.003). In APS II cases, all patients presented with PAI and AIT, with only one patient having T1DM. If PAI and AIT were not diagnosed simultaneously, the time interval between subsequent diagnoses ranged from 0.2 to 15.4 years. In 26.1% of the APS III patients, T1DM and AIT were diagnosed simultaneously, primarily due to AIT being screened in patients with newly suspected T1DM in the NTU and NTUH systems. However, when the two diseases were not diagnosed concurrently, the median latency time was 6.1 years from the diagnosis of T1DM to AIT, and 2.8 years from the diagnosis of AIT to T1DM.

**Table 2:** Comparison of patients with type II and III autoimmune polyendocrine syndromes. (*n* = 187). Data are presented as *n*, *n* (%), or as median (IQR).

	Type II	Type III	*P*^a^
*n* (%)	7 (3.7)	180 (96.3)	
Gender (M:F)	2:5	43:137	0.675
Age at APS diagnosis (years)	50.8 (29.9–65.5)	23.9 (13.8–36.3)	**0.003**
Diseases included in the diagnostic criteria of APS			
T1DM	1	180	
AIT	7	180	
PAI	7	0	
First diagnosis of T1DM, AIT, and PAI^b^			
T1DM (M:F)	0	109 (27:82)	
Age at diagnosis (years)		23.1 (14.3–34.4)	
AIT (M:F)	2 (0:2)	23 (0:23)	
Age at diagnosis (years)	50.8; 65.5	38 (26.9–50.6)	
PAI (M:F)	3 (1:2)	0	
Age at diagnosis (years)	29.9; 48.2; 57.4		
T1DM and AIT at almost the same time (M:F)	0	47 (16:31)	
Age at diagnosis (years)		15.6 (10.4–31)	
PAI and AIT at almost the same time (M:F)	2 (1:1)	0	
Age at diagnosis (years)	19.4; 72.7		
Time (years) from first to second diagnosis			
From T1DM to AIT		6.1 (3.2–10.9)	
From AIT to T1DM		2.8 (0.6–8.2)	
From AIT to PAI	0.7; 1.4		
From PAI to AIT	0.2; 2.7; 15.4		

^a^Comparison between patients with type II and type III APS; bold values denote statistical significance at the *P* < 0.05 level; ^b^Cannot be identified from chart review of one patient.

AIT, autoimmune thyroid disease; APS, autoimmune polyendocrine syndrome; F, female; M, male; PAI, primary adrenal insufficiency; T1DM, type 1 diabetes mellitus.

Table 3 provides a detailed overview of thyroid function status and the thyroid autoantibody concentrations. Among the patients with APS II, two individuals had hyperthyroidism, and four presented with hypothyroidism. In contrast, within the APS III group, 53 patients experienced hyperthyroidism, while 40 exhibited hypothyroidism. Nearly half of the APS III patients (48.3%) maintained a euthyroid state throughout the follow-up periods. These patients were significantly younger at the time of APS diagnosis compared to those with hyperthyroidism or hypothyroidism (*P* < 0.001 and *P* = 0.049, respectively). Furthermore, the thyroid autoantibody concentrations were lower in the group of patients with a euthyroid status. Their TBII levels were significantly lower than those of patients with hyperthyroidism (*P* < 0.001 after adjustment for sex and age at APS diagnosis). Additionally, their anti-TPO Ab levels were significantly lower than those in patients with hyperthyroidism and hypothyroidism (both *P* < 0.001 after adjustment for sex and age at APS diagnosis). The anti-Tg Ab levels were significantly lower than those of patients with hyperthyroidism and hypothyroidism (*P* = 0.019 and *P* = 0.006, respectively, after adjustment for sex and age at APS diagnosis).

**Table 3:** Thyroid function status and the thyroid autoantibody concentrations in patients with autoimmune polyendocrine syndrome (*n* = 187). Data are presented as *n* or median (IQR).

	Type II	Type III
Hyperthyroidism^a^	2	53
Overt	2	49
Subclinical	0	4
Age at APS diagnosis (years)	42.5 (19.4–65.5)	31 (17.7–41.4)
Gender (M:F)	0:2	7:46
TBII (%)	9.4; 12.5	58.9 (29–76.4)
Anti-TPO Ab (IU/mL)	85.4; 716.2	779.9 (212.7–1838)
Anti-Tg Ab (IU/mL)	3; 97.4	166 (13.1–420)
Hypothyroidism	4	40
Overt	2	15
Subclinical^b^	2	25
Age at APS diagnosis (years)	49.5 (39.1–54.1)	27 (13.4–35.9)
Gender (M:F)	1:3	12:28
TBII (%)	2.1^c^	8.1 (4.9–30.6)^d^
Anti-TPO Ab (IU/mL)	1035.5 (274.8–1819.9)	775.8 (164.4–2000)
Anti-Tg Ab (IU/mL)	137 (55–2064.5)	94.5 (23.9–669.6)
Euthyroidism	1	87
Age at APS diagnosis (years)	72.7	17 (12.6–28.8)^d,e^
Gender (M:F)	1:0	63:24
TBII (%)	20.8	3.9 (0.2–8)^d^
Anti-TPO Ab (IU/mL)	165.5	113.6 (29.3–638.2)^d,e^
Anti-Tg Ab (IU/mL)	79.9	21 (4–172.9)^d,e^

TBII reference range: <10%, TBII value ≤0.1% was labeled 0.1; Anti-TPO Ab reference range: <5.61 IU/mL, Anti-TPO AB value ≥2000 IU/mL was labeled as 2000; anti-Tg Ab reference range: < 14.3 IU/mL, Anti-Tg Ab value ≥10000 IU/mL was labeled 10,000 and Anti-Tg Ab value <3 IU/mL was labeled 3.

^a^Without non-iatrogenic hypothyroidism during observation; ^b^Five patients presenting subclinical hypothyroidism preceded by short-term hyperthyroidism; ^c^Available in only one patient; ^d^ *P* < 0.05 when compared with the hyperthyroidism group; ^e^ *P* < 0.05 when compared with the hypothyroidism group.

AIT, autoimmune thyroid disease; Anti-TPO Ab, anti-thyroid peroxidase antibody; APS, autoimmune polyendocrine syndrome; Anti-Tg Ab, anti-thyroglobulin antibody; TBII, thyrotropin-binding inhibitory immunoglobulin.

**Figure 1:**
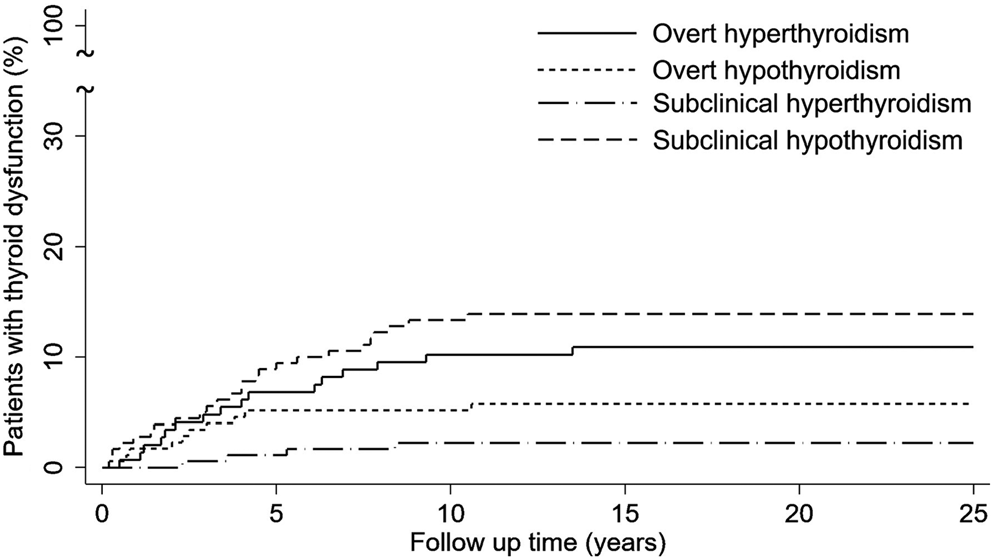
The time sequence of the occurrence of thyroid dysfunction in patients who had autoimmune thyroid disease and were in euthyroid status at diagnosis of APS III (*n* = 142).

In APS III patients, we observed that 21.1% developed thyroid dysfunction before the detection of thyroid autoantibodies (which means thyroid dysfunction was noticed first and autoantibodies were checked later to investigate the etiology of thyroid dysfunction), while the remaining patients developed thyroid dysfunction after positive thyroid autoantibodies were detected. Among the 49 patients who had overt hyperthyroidism, 16 developed hyperthyroidism after positive thyroid autoantibodies were detected, with a median latency time of 4.6 years (IQR: 1.8–6.6 years). Among the 15 patients with overt hypothyroidism, ten developed hypothyroidism after positive thyroid autoantibodies were detected, with a median latency time of 2.4 years (IQR: 0.8–3.8 years). The time sequence and the occurrence of thyroid dysfunction in patients with euthyroid status when positive thyroid autoantibodies were detected are shown in Fig. 1.

In patients with APS-III who developed overt thyroid dysfunction after AIT was diagnosed, the logistic regression model showed a positive correlation between age at diagnosis and the likelihood of developing overt thyroid dysfunction. For every year older patients diagnosed with APS III, there was a 3.6% increase in the risk of developing hyperthyroidism (OR: 1.036, 95% CI = 1.013–1.060, *P* = 0.002) and a 3.7% increase in the risk of developing hypothyroidism (OR: 1.037, 95% CI = 1.002–1.072, *P* = 0.035).

Among patients with APS II and APS III, serum APCA levels were available in 66 patients, 14 (21.2%) of whom were positive (Table 4). After excluding two patients who had anemia apparently unrelated to APS (both were thalassemia), we analyzed the correlation between APCA status and anemia. We found that the prevalence of anemia was significantly higher in the APCA-positive group than in the APCA-negative group (*P* < 0.001). Among the seven patients with anemia in the APCA-positive group, five patients were confirmed to have iron deficiency anemia (IDA), and one patient was confirmed to have both IDA and pernicious anemia caused by vitamin B12 deficiency. One patient had microcytic anemia without further investigation. As to the patient with anemia in the APCA-negative group, she was confirmed to have IDA.

**Table 4:** Anti-parietal cell antibodies (APCA) in patients with autoimmune polyendocrine syndromes (*n* = 66).

	Positive	Negative	*P*
APCA checked, *n* (%)	14 (21.2%)	52 (78.8%)	
Gender (M:F)	3:11	19:33	0.287
Age at APS diagnosis (years)	14.2 (11.5–23.1)	13 (8.6–16.3)	0.081
Anemia,^a^*n* (%)	7 (50%)	1 (2%)	**<0.001**

^a^Excluding other diseases causing anemia, such as thalassemia and hemolytic anemia. Bold values denote statistical significance at the *P* < 0.05 level.

## Discussion

Our study demonstrated the demographic, clinical, and serological characteristics of APS II and APS III patients in the Asian population. Compared with the Western population, a lower prevalence of patients with APS II was observed, and the peak age at APS diagnosis was relatively younger. Additionally, it showed that older age at diagnosis of APS was associated with a higher risk of developing overt thyroid dysfunction. Positive APCA was associated with a higher risk of anemia in patients with APS III.

To our knowledge, our research represents the only large-scale study of APS II and APS III in Asia (16, 17, 18, 19, 26, 27), with previous research primarily focusing on adult APS types conducted in Western countries (5, 13, 15, 28, 29, 30). The comparison of studies on adult APS types is summarized in Table 5, but the inclusion criteria varied among these studies. The first large-scale case series focusing on APS II was reported in the USA in 1981 (13). Researchers found that women had a higher incidence than men across all age groups, and the age of onset varied widely from childhood to late adulthood and peaked in midlife (13). Our study also observed a higher incidence in women, with the median age at APS II diagnosis being 50.8 years old. Another large-scale case series conducted in Germany in 2003 encompassed all three adult types of APS (5). Their results showed that T1DM and AIT were the most common combinations, followed by PAI and AIT (5). In both studies, T1DM was the initial presentation in about half of the patients, with variable time intervals between the two autoimmune endocrinopathies, from months to years. If AIT was the first component, the time interval until the next immune-endocrinopathy was shorter (5, 31). Our study of APS III also showed that T1DM was the most common first presentation, and the diagnostic interval from AIT to T1DM was also shorter than that from T1DM to AIT when not diagnosed concurrently (*P* < 0.001). Regarding the literature on the Asian population, two prior studies focusing on APS III were reported from Japan and China with a limited number of participants (*n* = 54 and 36, respectively) (26, 27). A study in Taiwan attempted to study all three adult types of APS, but no APS II patients were recruited (17). Our study sought to recruit patients with APS II and APS III. The results showed that APS II was particularly rare, accounting for only 3.7% of all study participants. This percentage was much lower than in the Western population, which was approximately 17–19% (5, 29). The observed difference may be genuine. However, it could also be caused by the lack of evaluation of adrenal-specific autoantibodies, considering a significant portion of our study subjects were initially identified from T1DM patients by antibody screening. Another difference in race is diagnostic age. In the West, the peak incidence of initial diagnosis of adult-type APS was 20–60 years old, mostly in the third or fourth decade of life (32). In our study, the peak age of APS diagnosis was in the second decade, followed by the third decade. The reasons for these disparities remain unclear. One possibility is that APS II is diagnosed later in life than APS III, usually about the fourth decade of life, and is less prevalent in the Asian population. It is also noteworthy that some patients with APS III may later shift to APS II if the follow-up period is sufficiently long. Another possible reason is the routine screening of thyroid autoantibodies in most patients with T1DM and PAI in our hospital system, potentially leading to an earlier diagnosis age for APS due to such screening practices.

**Table 5:** Comparison of studies of adult autoimmune polyendocrine syndrome (APS).

	Patients, *n*	Female/male	Age at APS diagnosis (years)	Region	PAI (%)	AIT (%)	T1DM (%)
Neufeld *et al.* (13)^a^	224	1.8	NA	USA	100	69	52
Papadopoulos *et al.* (28)^a^	22	2.67	35 (18–61)	Sweden	100	72.7	40.9
Dittmar *et al.* (5)^b^	151	3.0	NA	Germany	18.5	65.6	60.9
Betterle *et al.* (15)^a^	146	4	NA	Italy	100	88	23
Hung *et al.* (17)^b^	19	1.38	NA	Taiwan	0	100	79
Storz *et al.* (29)^b^	75	1.88	NA	Germany	17.3	94.7	65.3
Horie *et al.* (26)^c^	54	4.4	NA	Japan	0	100	100
Ben-Skowronek *et al.* (30)^c^	67	3.19	NA	Poland	0	100	100
Zhao *et al.* (27)^c^	36	2	48.0 ± 16.2	China	0	100	100
Our study^b^	187	3.16	24.1 (14.0–37.6)	Taiwan	3.7	100	96.8

^a^Only patients with APS II (PAI and either T1DM or AIT) were included; ^b^Patients with adult APS (autoimmune diseases involving at least two endocrine organs) were included; ^c^Only patients with T1DM and AIT were included.

APS, autoimmune polyendocrine syndrome; AIT, autoimmune thyroid disease; PAI, primary adrenal insufficiency; NA, not available; T1DM, type 1 diabetes mellitus.

Gender differences among APS patients were identified in our study. Females predominated with a ratio of 3:1, similar to other studies (2, 31, 32). Additionally, men tended to be diagnosed earlier in life than women. Although T1DM was the most common initial disease manifestation in both sexes, a higher proportion of female patients presented with AIT as their first disease manifestation compared to male patients. The exact cause of this gender disparity remains unknown, but it is probably related to sex hormones and genetic background.

AIG was found in 4–10% of patients with adult-type APS (2). It is a chronic gastric inflammatory disease characterized by positive APCA, leading to malabsorption of essential elements and may cause IDA and pernicious anemia due to vitamin B12 deficiency (25). Our study showed that positive APCA was significantly associated with anemia in patients with APS III, with a prevalence of 54.5%. Therefore, we suggest that APCA should be checked in patients with APS, especially those with anemia.

The presence of an autoimmune endocrine disease is usually associated with a higher risk of developing autoimmunity in other tissues (2, 32). In a meta-analysis of 293,889 T1DM patients, 9.8% had hypothyroidism, 4.5% had celiac disease, 4.3% had gastric autoimmunity, and 0.2% had adrenal insufficiency (33). Autoimmune adrenal insufficiency is frequently accompanied by at least one other autoimmune disorder, with primary hypothyroidism being the most prevalent, followed by vitiligo, gonadal failure, Graves’ disease, pernicious anemia, T1DM, and celiac disease (34). Therefore, it is crucial to conduct serological screening for autoantibodies in patients with monoglandular autoimmune disease to evaluate the risk of APS, especially in those with PAI or T1DM (2). Additionally, families of APS patients often exhibit a high prevalence of several autoantibodies (5). Studies have shown that approximately one-seventh of first-degree relatives of patients with adult-type APS have an unrecognized endocrine disorder (31, 35). It is essential to perform functional testing of endocrine glands in autoantibody-positive patients, and their first-degree relatives should also undergo serological screening (2, 36, 37). Furthermore, autoantibodies identified via screening may precede overt clinical presentation over several years. Reports indicate a prolonged phase of up to 20 years between the autoantibodies detection and the onset of overt glandular dysfunction (28). In a prospective cohort study, patients with multiple islet autoantibodies had a 69.7% progression rate to T1DM at the 10-year follow-up, while those with single islet autoantibodies had a 14.5% progression rate (38). The cumulative incidence of adrenal insufficiency was 24.6% in adult-type APS patients with positive 21-hydroxylase autoantibodies during a median follow-up of 10 years (39). The time interval between the detection of autoantibodies and the presentation of the disease is usually longer in AIT (31). Our study revealed that more patients with APS III developed hyper- or hypothyroidism when diagnosed with APS at older ages. These patients might benefit from regular follow-up to prevent potential critical events like adrenal crises or other chronic complications. Hence, routine follow-up every 2–3 years is crucial for adult-type APS patients and their relatives (2, 5).

Our study has some limitations. First, this was a retrospective study, in which the prevalence of APS and the time sequence of each organ involved could not be accurately assessed. Second, tests for adrenal antibodies, such as a 21-hydroxylase antibody, were not available in our hospital system. Furthermore, this study did not have a reliable family history of autoimmune endocrinopathies and associated genetic profiles. Such issues should be addressed in future research.

Nevertheless, our study demonstrated the comprehensive demographic, clinical, and serological features of patients with APS II and APS III in Taiwan. A second autoimmune endocrine disease may manifest simultaneously or years after the first glandular failure. Early detection of specific autoantibodies and long-term follow-up of endocrine function is recommended for patients with APS and their families to prevent disease complications.

## Declaration of interest

The authors declare that there is no conflict of interest that could be perceived as prejudicing the impartiality of the study reported.

## Funding

This work was supported by the Liver Disease Prevention and Treatment Research Foundation, Taiwan; National Taiwan Universityhttp://dx.doi.org/10.13039/501100006477 Hospital; National Taiwan Universityhttp://dx.doi.org/10.13039/501100006477; and Wong-Yuan Endocrine Fund in Taiwan.

## Ethical approval

Ethical approval was obtained from the Ethics Committee of National Taiwan University Hospital (protocol number: 202112250RINC). This study was registered at ClinicalTrials.gov (ID: NCT05578105).
